# Perchlorate-Reducing Bacteria from Hypersaline Soils of the Colombian Caribbean

**DOI:** 10.1155/2019/6981865

**Published:** 2019-02-17

**Authors:** Rosa Acevedo-Barrios, Angela Bertel-Sevilla, Jose Alonso-Molina, Jesus Olivero-Verbel

**Affiliations:** ^1^Environmental and Computational Chemistry Group, School of Pharmaceutical Sciences, Zaragocilla Campus, University of Cartagena, Cartagena 130015, Colombia; ^2^Biological and Chemical Studies Group, School of Basic Sciences, Technological University of Bolívar, Cartagena 130010, Colombia; ^3^Research Institute of Water and Environmental Engineering, Universitat Politècnica de València, Valencia 46022, Spain

## Abstract

Perchlorate (ClO_4_^−^) has several industrial applications and is frequently detected in environmental matrices at relevant concentrations to human health. Currently, perchlorate-degrading bacteria are promising strategies for bioremediation in polluted sites. The aim of this study was to isolate and characterize halophilic bacteria with the potential for perchlorate reduction. Ten bacterial strains were isolated from soils of Galerazamba-Bolivar, Manaure-Guajira, and Salamanca Island-Magdalena, Colombia. Isolates grew at concentrations up to 30% sodium chloride. The isolates tolerated pH variations ranging from 6.5 to 12.0 and perchlorate concentrations up to 10000 mg/L. Perchlorate was degraded by these bacteria on percentages between 25 and 10. 16S rRNA gene sequence analysis indicated that the strains were phylogenetically related to *Vibrio*, *Bacillus*, *Salinovibrio*, *Staphylococcus*, and *Nesiotobacter* genera. In conclusion, halophilic-isolated bacteria from hypersaline soils of the Colombian Caribbean are promising resources for the bioremediation of perchlorate contamination.

## 1. Introduction

Perchlorate pollution is a problem of global impact because it has a negative effect on ecosystems with a loss of environmental quality, which increases with anthropogenic activity. Perchlorate is an ubiquitous emerging contaminant produced from both natural and anthropogenic sources [1], particularly present in areas associated with the use and manufacture of rockets and ammunition. This compound is a potent endocrine disruptor that mainly affects the fixation of iodine by the thyroid gland, responsible for regulating metabolism, growth, and development [2, 3], thus being dangerous to infants and young children [4]. Acute, short-term exposure has been shown to affect the nervous, respiratory, immune, and reproductive systems [5]. It has also been related to thyroid cancer and teratogenesis during the first trimester of pregnancy [6].

Perchlorate is highly distributed in ecosystems and organisms; thus, it is frequently found in several matrices, including breast milk, fertilizers, plants, food, and human tissues. This scenario has led to the prioritization of studies that allows for the removal of this contaminant from polluted sites, as it is extremely toxic and persistent; therefore, efficient treatments for its degradation are needed in order to maintain the quality of soils from biodiversity hotspots. There are different physicochemical techniques commonly used for the environmental removal of this anion, such as ion exchange, but it is not selective and the process usually only separates the perchlorate from contaminated sources [7], also generating by-products, which requires subsequent treatment [7]. Perchlorate is persistent but possesses biodegradability [8]. However, enzymes such as the perchlorate reductase and superoxide chlorite carry out the reduction or elimination of perchlorate. A reductase enzyme is responsible for reducing perchlorate to chlorate and chlorate to chlorite, while the enzyme superoxide chlorite changes chlorite to chloride and molecular oxygen. Biological reduction through the use of bacteria completely degrades the perchlorate ions into Cl^−^ and O_2_ (equation (1)) [9–11]. The perchlorate degradation pathway is as follows:

(1) $$\begin{array}{c} {{\text{ClO}}_{4}}^{-}\left(\text{perchlorate}\right)⟶\left({{\text{ClO}}_{3}}^{-}\right)\left(\text{chlorate}\right)⟶\left({{\text{ClO}}_{2}}^{-}\right)\left(\text{chlorite}\right)⟶{\text{Cl}}^{-}\left(\text{chloride}\right)+{\text{O}}_{2} \end{array}$$

Marine soils usually contain bacterial species with biochemical versatility and ability to tolerate salt, being an interesting target for researchers due to the potential reduction of environmental perchlorate [10]. The reason for selecting this type of environment is that degradation of perchlorate may be carried out using salt-tolerant bacteria [12], although this perchlorate-reduction process could be impaired with increasing salinity [11, 13]. Moreover, these organisms are available in diverse environments, from Antarctica, saline lakes, and hot springs, even in hyperthermophilic and hypersaline soils [10, 11]. Additionally, perchlorate deposits in these environments may be formed by chemical reactions between sodium chloride from land or sea and ozone [14].

Pilot testing of biotechnologies using perchlorate-reducing bacteria has been studied and tuned in suspension, fixed-bed, fluidized, and biofilm reactors [15, 16], evaluating the effectiveness of treatments on soil and water contaminated with perchlorate [15]. Nowadays, the use of integrated systems, combining physicochemical treatments and perchlorate-reducing halophilic bacteria, is being studied to increase the efficiency of water treatment, allowing the handling of residual flows with high salinity and large perchlorate concentrations, simultaneously solving the waste disposal problem [17].

Colombia has a variety of ecosystems with different climatic and biogeochemical features, which facilitates the development of different halophilic bacteria. The aim of this study was the characterization of perchlorate-reducing bacteria recovered from hypersaline soils of the Colombian Caribbean.

## 2. Materials and Methods

### 2.1. Study Area and Sample Collection

**Figure 1:**
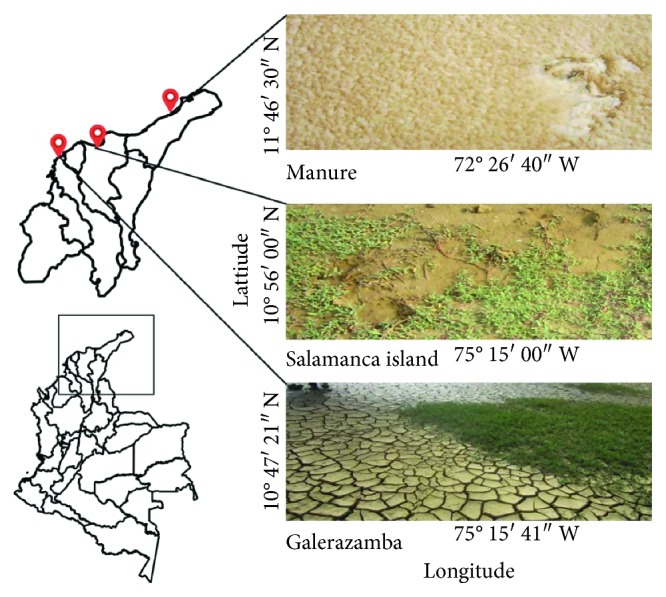
Map of the Caribbean region of Colombia showing the geographic location of the sampling sites.

Soil samples were obtained from salt mines of Colombia, specifically from Galerazamba-Bolivar (10°47′21″N, 75°15′41″W), Manaure-Guajira (11°46′30″N, 72°26′40″ W), and saline soils located in Salamanca Island-Magdalena (10°56′00″N, 75°15′00″W) (Figure 1). A sterile spatula was employed to collect approximately 100 g of soil from the upper 1–10 cm layer in May 2015. All samples were collected in 50 mL Falcon tubes, kept refrigerated at 4°C, and taken to the laboratory for processing. Salinity and pH were recorded for each sample according to Nozawa-Inoue et al. [18].

### 2.2. Isolation of Strains and Culture Conditions

Isolation, purification, and preservation techniques were used as described by Shimkets and Rafiee [19]. Samples were treated with amphotericin B (0.25 mg/mL) for 3 h until inoculation (50 *µ*L) in the isolation media. Subsequently, samples were incubated in Petri dishes containing modified sterilized agar Luria-Bertani in seawater (LB NaCl) [20] and incubated at 37°C for 24 h under aerobic conditions [21]. Bacterial growth was monitored by observation of colonies. For preservation, a bacterial colony was transferred to modified LB broth and incubated at 37°C aerobically during 12 h, adjusting the cell density to 0.5 Mc-Farland turbidity standard. Then, 720 *µ*L of each culture was transferred to a Cryovial with 80 *µ*L glycerol and stored at −80°C.

### 2.3. Molecular Identification

#### 2.3.1. 16S rRNA Gene Sequencing and Phylogenetic Analysis

For 16S rRNA gene amplification, genomic DNA was extracted using a QIAamp® DNA Mini Kit (Qiagen, CA, USA) as described by the manufacturer. The 16S rRNA gene was amplified from the total genomic DNA of the bacterial strains by PCR using the forward primer PF (5′-AGAGTTTGATCCTGGCTCAG-3′) and the reverse primer 1492R (5′-ACCTTGTTACGACTT-3′) [22, 23]. PCR was performed with an AmpliTaq Gold® 360 Master Mix (Applied Biosystems) according to the manufacturer's instructions. Each 25 *μ*L reaction mixture contained 0.4 *μ*M of each primer and ∼100 ng of template DNA. The amplification was performed as follows: initial denaturation for 10 min at 95°C, 25 cycles of denaturation for 1 min at 94°C, annealing for 1 min at 43°C, and extension for 1.5 min at 72°C, and a final extension for 5.5 min at 72°C. All PCR products were checked by electrophoresis on 1.2% (w/v) agarose gels stained with ethidium bromide (10 mg/mL) and analyzed using a gel documentation system (IngGenius 3 System-Syngene).

The PCR product was purified with a QIAquick PCR purification kit (Qiagen, CA, USA), following the manufacturer's instructions. Automated DNA sequencing was performed by the National Center for Genomic Sequencing (CNSG) (Medellin-Colombia) using PF and 1492R primers. Sequence readings were edited and assembled with the CAP3 software [24]. The resulting 16S rRNA gene sequences were compared to reference strains with validly published names using the EzTaxon-e server (http://www.ezbiocloud.net/eztaxon). After multiple alignments of the data via CLUSTAL_W, four methods, including neighbor joining (NJ) [25], maximum likelihood (ML) [26], minimum evolution (ME), and maximum parsimony (MP) [27], were used to perform phylogenetic analysis. Phylogenetic trees were constructed using MEGA version 6 [28]. Distances were calculated using the Kimura correction in a pair-wise deletion manner [29]. The topologies of the phylogenetic tree were evaluated by the bootstrap resampling method described by Felsenstein [30] with 1000 replicates. The GenBank/EMBL/DDBJ accession numbers for the 16S rRNA gene sequences of isolated strains BBCOL-031, BBCOL-023, BBCOL-024, BBCOL-025, BBCOL-026, BBCOL-027, BBCOL-028, BBCOL-029, BBCOL-032, and BBCOL-033 are KX821664–KX821673, respectively.

### 2.4. Morphological Characterization

Strains were incubated on LB NaCl agar, and both growth and morphogenesis were observed under light microscopic examination (Olympus microscope BX41). Gram staining was conducted following Bergey's Manual Taxonomic Key [31] and Koneman's Microbiologic Atlas [32]. Samples were further processed by means of scanning electron microscopy (SEM) visualization as previously described [33].

### 2.5. Biochemical Characterization

Biochemical characteristics were investigated using the BBL Crystal™ Kit (Becton Dickinson Microbiology Systems, Cockeysville, USA) as described by the manufacturer. Catalase and oxidase tests were performed according to the reported methods [21].

### 2.6. Chloride Susceptibility Assay

All strains were assayed for perchlorate susceptibility in LB broth in the presence of NaCl (3.5, 5.0, 7.5, and 30% w/v) [20, 34]. The experiments started adding 20 *µ*L of cell suspension, OD = 0.6, into 5 mL LB broth. The cultures were incubated at 37°C for 24 h, and after that, strains showing turbidity [35] were identified as resistant to NaCl. The experiments were carried out by triplicates and performed three times.

### 2.7. Perchlorate Susceptibility Assay

All strains were assayed for perchlorate susceptibility in LB broth in the presence of perchlorate at concentrations of 100, 250, 500, 750, 1000, 1250, 1500, 2000, 3000, 5000, 7500, and 10000 mg/L [20, 36, 37]. The experiments were carried out as described for the chloride susceptibility assay. After the incubation time (24 h), a culture of each isolate was tested on LB agar at their corresponding KClO_4_ concentrations to confirm cell viability and purity of each of the bacterial strains.

### 2.8. Evaluation of Perchlorate Reduction by Isolates

The experiments were carried out using concentration of 10000 mg/L KClO_4_ in LB with 3.5% NaCl, inoculating with the isolates as described in the chloride susceptibility assay and incubating for a 24-hour time period at 37°C. After incubation time, the final KClO_4_ concentration was measured with a Thermo Scientific Orion 93 perchlorate electrode (Thermo Fisher Scientific Inc., Beverly, MA), used according to the manufacturer's instructions. The difference in concentration after and before the incubation was employed to calculate the perchlorate reduction percentage elicited by each strain.

## 3. Results and Discussion

### 3.1. Molecular Identification

Almost-complete 16S rRNA gene sequences were obtained from strains BBCOL-023 to BBCOL-033 (GenBank accession numbers: KX821664–KX821673). The results of the phylogenetic analysis showed that these strains belonged to members of *Bacillus*, *Vibrio*, *Salinivibrio*, *Nesiotobacter*, and *Staphylococcus*. Strain BBCOL-023 presented 99% similarity with *Nesiotobacter*; strains BBCOL-024, BBCOL-028, BBCOL-029, and BBCOL-033 had 99% homology with *Bacillus* species; and strains BBCOL-025, BBCOL-026, BBCOL-027, and BBCOL-031 had 99% homology with members of the Vibrionaceae family, particularly *Vibrio* and *Salinivibrio* species. Strain BBCOL-032 presented 99% similarity to *Staphylococcus* spp.

The 16S rRNA gene sequence had similar values between strains BBCOL-024, BBCOL-028, BBCOL-029, and BBCOL-033, and validly named type strains of *Bacillus* were calculated by using the EzTaxon-e server. Strains BBCOL-024 and BBCOL-028 showed high 16S rRNA gene sequence similarities with *Bacillus vallismortis* DV1-F-3(T) (99.6 and 99.5%, respectively), *Bacillus subtilis* subsp. *inaquosorum* KCTC 13429(T) (99.6 and 99.4%, respectively), and *Bacillus subtilis* subsp. *spizizenii* NRRL B-23049(T) (99.6 and 99.4%, respectively).

**Figure 2:**
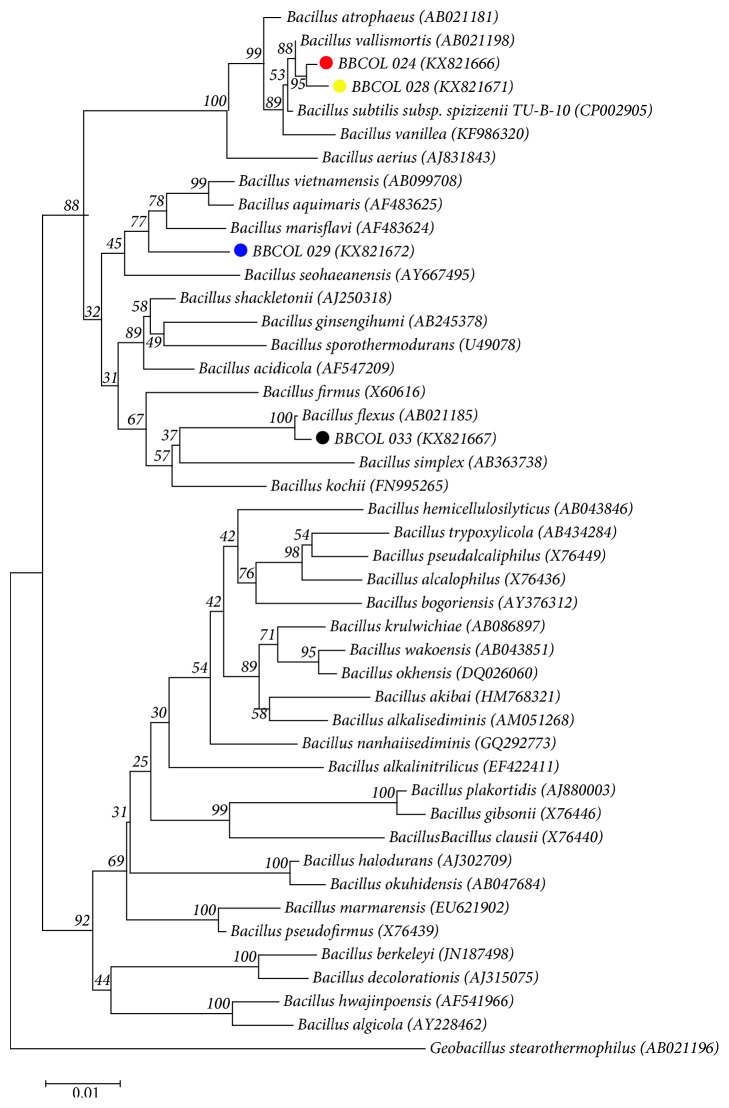
Neighbor joining tree based on 16S rRNA gene sequences showing the phylogenetic position of strains BBCOL-024, BBCOL-028, BBCOL-029, and BBCOL-033 compared to the most closely related members of the genus *Bacillus.* Bootstrap values based on 1000 replications are listed as percentages at the branching points. Accession numbers are given in parentheses. Bar, 0.01 nucleotide substitutions per nucleotide position.

Levels of 16S rRNA gene sequence similarity between strains BBCOL-024 and BBCOL-028 and other current members of the genus *Bacillus* were below 99.0%. In the neighbor-joining (Figure 2) and the minimum-evolution phylogenetic dendrograms based on 16S rRNA gene sequences, strains BBCOL-024 and BBCOL-028 were placed in a cluster in *Bacillus* and were shown to be closely related to *B. vallismortis*, *B. subtilis* subsp. *spizizenii* TU-B-10, *B. vanillea*, and *B. atrophaeus*.

Strain BBCOL-029 shares high-sequence similarity with *B. oryzaecorticis* R1 (T), *B. haikouensis* C-89(T), *B. aquimaris* TF-12(T), and *B. vietnamensis* 15-1(T) with 98.2, 97.9, 97.9, and 97.9% respectively, and nucleotide differences of 20, 29, 30, and 29 nucleotides, respectively. The comparative analysis of 16S rRNA gene sequences and phylogenetic relationships showed that the BBCOL-029 strain lies in a subclade in the tree with *B. marisflavi, B. aquimaris*, and *B. vietnamensis* (supported by a bootstrap value of 77% (Figure 2)), with which it shares the highest 16S rRNA gene sequence similarity. The affiliation of strain BBCOL-029 and its closest neighbors was also supported by the maximum parsimony and maximum likelihood algorithms with bootstrap values above 70%. EzTaxon-e server search analysis revealed that the BBCOL-033 strain is closely related to *B. flexus* FO 15715(T) (99.7%, 16S rRNA gene sequence similarity), *B. paraflexus* RC2 (T) (99%), *B. megaterium* NBRC 15308 = ATCC 14581(T) (98.5%), and other bacilli (<97%). The sequence similarities of strain BBCOL-033 with *B. flexus* using different clustering algorithms (100% in NJ tree; 100% in ME tree; and 100% in ML tree) along with the EzTaxon-e server analysis consistently indicated that *B. flexus* is the closest relative.

**Figure 3:**
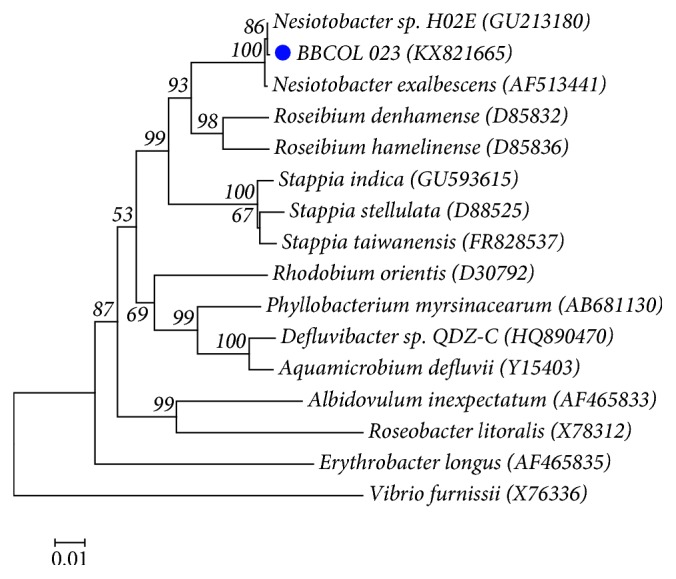
Neighbor joining phylogenetic tree based on 16S rRNA gene sequences showing the relationships of strain BBCOL-023 and related taxa. Percentage bootstrap values based on 1000 replications are given at branch points. The gammaproteobacterium *Vibrio furnissii* (X76336) was used as the outgroup. Bar, 0.01 substitutions per nucleotide position.

For strain BBCOL-023, 1329 nt of the 16S rRNA gene sequence was determined. Comparative 16S rRNA gene sequence analysis showed that strain BBCOL-023 was most closely related to members of the genus *Nesiotobacter.* Strain BBCOL-023 shares the highest sequence similarity with *Nesiotobacter exalbescens* LA33B (T), *Roseibium aquae* DSG-S4-2 (T), and *Pseudovibrio hongkongensis* UST20140214-015B (T) with 99.8, 95.9, and 95.7% and nucleotide differences of 3, 55, and 57, respectively. The 16S rRNA gene sequence similarities to the type strains of other members of the family Rhodobacteraceae with validly published names were below 94%. In the phylogenetic tree based on the NJ algorithm (Figure 3), strain BBCOL-023 formed a single clade with *Nesiotobacter exalbescens* (supported by a bootstrap value of 100% (Figure 3)), with which it shares the highest 16S rRNA gene sequence similarity. The affiliation of strain BBCOL-023 and its closest neighbors was also supported by the MP and ML algorithms with above 90% bootstrap values.

**Figure 4:**
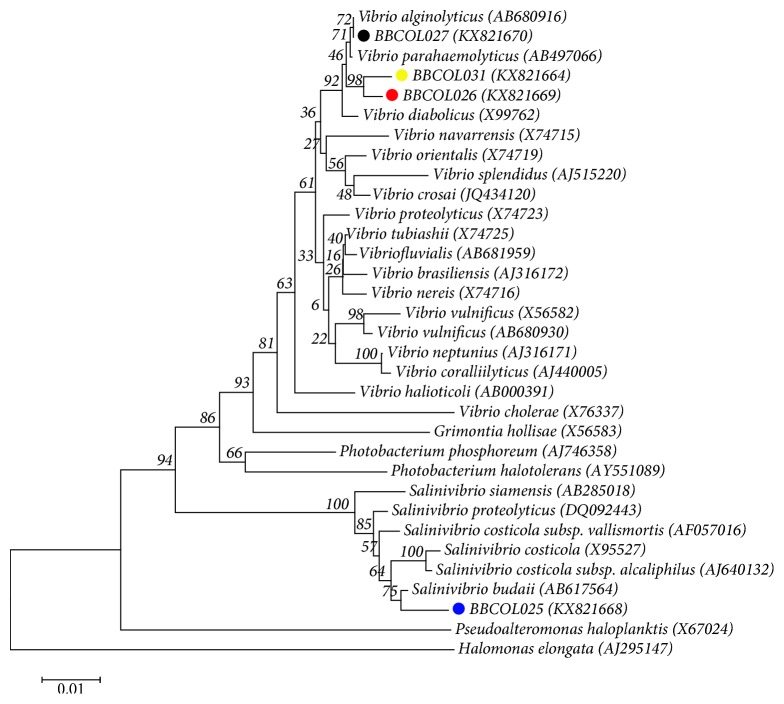
Neighbor joining tree based on 16S rRNA gene sequences showing the phylogenetic position of strains BBCOL-025, BBCOL-026, BBCOL-027, and BBCOL-031 compared to the most closely related members of the Vibrionaceae family. Bootstrap values based on 1000 replications are listed as percentages at the branching points. Accession numbers are given in parentheses. Bar, 0.01 nucleotide substitutions per nucleotide position.

The 16S rDNA sequences of strains BBCOL-025, BBCOL-026, BBCOL-027, and BBCOL-031 determined in this study comprised 1402, 1361, 1399, and 1389 nt, respectively, representing approximately 90% of the *Escherichia coli* 16S rRNA sequence. The results of phylogenetic analysis of the 16S rRNA gene sequences revealed that the isolated strains were related phylogenetically to members of the Vibrionaceae family and belong within the phyletic group classically defined as the genus *Salinivibrio* and *Vibrio* (Figure 4). Strain BBCOL-025 shows high 16S rRNA gene sequence similarities with *Salinivibrio costicola* subsp. *vallismortis* DSM 8285(T) (98.4%), *Salinivibrio costicola* subsp. *costicola* ATCC 33508(T) (97.8%), and *Salinivibrio proteolyticus* AF-2004(T) (97.7%). Levels of 16S rRNA gene sequence similarity between strain BBCOL-025 and the other current members of the genus *Salinivibrio* are below 97%. In the neighbor joining (Figure 4) and the minimum evolution phylogenetic dendrograms based on 16S rRNA gene sequences, strain BBCOL-025 was placed in a cluster in the *Salinivibrio* genus and was shown to be closely related to *S. budaii* and *S. costicola* subsp. *alcaliphilus* (supported by a bootstrap value of 75%). 16S rRNA gene sequence comparison between the BBCOL-026 strain and other members from the *Vibrionaceae* family by using the EzTaxon-e server indicated that the strain was closely related to members of *Vibrio* genus, showing 99.2% gene sequence similarity to *V. antiquarius* Ex25 (T), 99% to *V. alginolyticus* NBRC 15630(T), 98% to *V. neocaledonicus* NC470 (T), and 98.9% to *V. natriegens* DSM 759(T). Likewise, strains BBCOL-027 and BBCOL-031 show high 16S rRNA gene sequence similarities with *V. alginolyticus* NBRC 15630(T) (99.8 and 98.9%, respectively) and *V. antiquarius* Ex25(T) (99.5 and 98.9%, respectively). A phylogenetic tree, generated from the neighbor joining algorithm, showed that strains BBCOL-026 and BBCOL-031 both fell within the radiation of the cluster comprising *Vibrio* species and formed a coherent cluster that is supported by a bootstrap analysis at a confidence level of 98% (Figure 4). This cluster joins the phylogenetic clade comprising *V. alginolyticus* and *V. parahaemolyticus*, which is supported by a 71% bootstrap value. This topology was also found in trees generated with the ML and MP algorithms (not shown). The NJ and ME phylogenetic trees based on 16S rRNA gene sequence data showed that the strain BBCOL-027 forms a coherent cluster with *Vibrio alginolyticus* (a bootstrap value of 72%).

**Figure 5:**
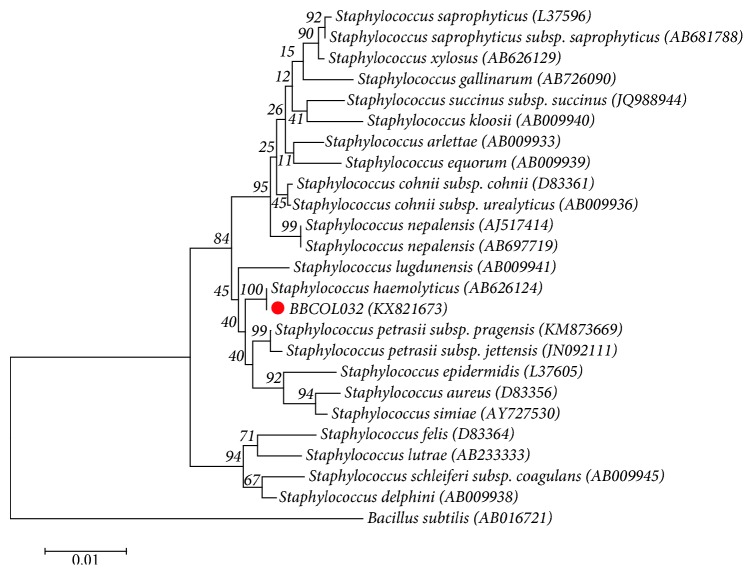
Neighbor joining phylogenetic tree based on 16S rRNA gene sequences showing the relationships of strain BBCOL-032 and related taxa. Percentage bootstrap values based on 1000 replications are given at branch points. *Bacillus subtilis* (AB016721) was used as the outgroup. Bar, 0.01 substitutions per nucleotide position.

For strain BBCOL-032, 1416 nt of the 16S rRNA gene sequence was determined. Comparative 16S rRNA gene sequence analysis showed that strain BBCOL-032 was more closely related to the *Staphylococcus* species. Strain BBCOL-032 shares highest sequence similarity with *Staphylococcus haemolyticus* ATCC 29970(T) (99.9%), *Staphylococcus petrasii* subsp. *pragensis* NRL/St 12/356(T), and *Staphylococcus petrasii* subsp*. jettensis* SEQ110 (T) (99.2%). The 16S rRNA gene sequence similarities to strains from other members of the Staphylococcaceae family were below 99%. In the phylogenetic tree based on the NJ algorithm (Figure 5), strain BBCOL-032 fell within a coherent cluster comprising *S. haemolyticus*, *S. petrasii* subsp. *pragensis*, *S. petrasii* subsp. *jettensis*, and *S. lugdunensis*. The sequence similarities of strain BBCOL-032 with *S. haemolyticus* using different clustering algorithms (100% in NJ tree; 99% in ME tree; and 100% in ML tree) along with the EzTaxon-e server analysis consistently indicated that *S. haemolyticus* is the closest relative.

### 3.2. Microscopic and Biochemical Characterization

**Figure 6:**
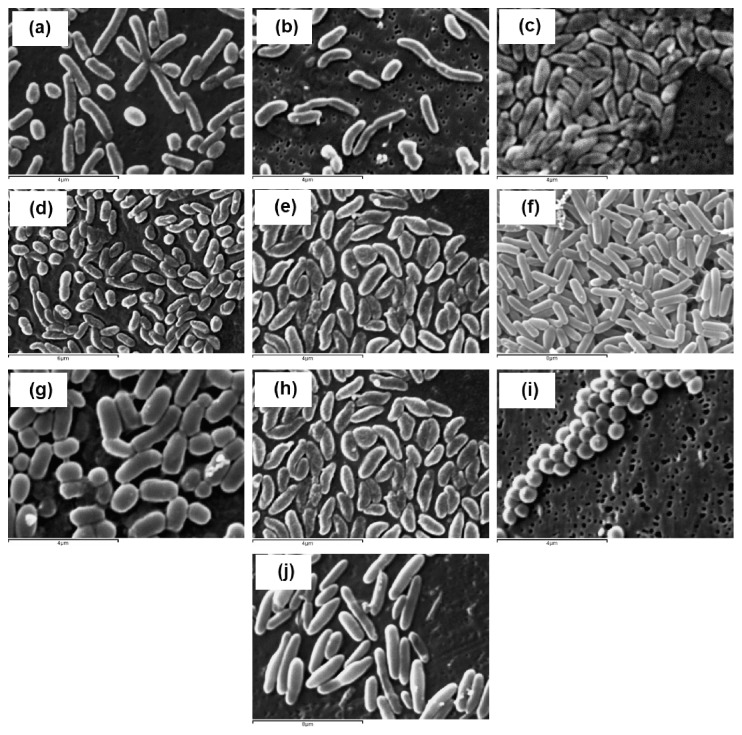
Cell morphology studied by SEM. Bacteria isolated from hypersaline soils: (a) BBCOL-023; (b) BBCOL-024; (c) BBCOL-025; (e) BBCOL-027; (f) BBCOL-028; (g) BBCOL-029; (h) BBCOL-031; (i) BBCOL-032; (j) BBCOL-033 (SEM at 10000x).

The colonies of strains (BBCOL-023 to BBCOL-033) on LB agar were circular, convex, and smooth. Cells were facultative anaerobic, with an optimal growth at pH 6.5 to 7.5. Morphological features are observed by SEM (Figure 6), and biochemical characteristics of isolated bacteria strains are shown in Table 1. Morphological and biochemical characteristics detected in the isolated strain BBCOL-023 correspond to the species *Nesiotobacter*, as previously reported [38]. The strains were able to utilize lactose, mannose, galactose, sorbitol, and sucrose as carbon sources. Nitrate was reduced to nitrite. The strains were ONPG- and H_2_S-negative.

Microscopic morphological results showed that isolates BBCOL-024, BBCOL-028, BBCOL-029, and BBCOL-033 correspond to the genus *Bacillus* [39]. When grown at 37°C during 24 h in LB, the cells were Gram-positive bacilli, with a size between 0.6-0.7 *μ*m and 1.6-1.7 *μ*m. Endospores were ellipsoidal. Cells were motile, aerobic, facultative anaerobic, and oxidase- and catalase-positive. Optimal growth conditions of BBCOL-024 were at pH 6.0–6.5 and 37°C. These isolates showed Voges–Proskauer and nitrate reduction activity. However, they were ONPG- and H_2_S-negative.

The cells of strain BBCOL-025, *Salinovibrio costicola*, were Gram-negative, motile, nonsporulating, and curved, presenting an average bacterial size of 1.4 × 0.7 *µ*m. In addition, these were motile, facultative anaerobic, and oxidase- and catalase-positive. Optimal growth conditions for BBCOL-025 were at pH 6.0–6.5 and 37°C. The isolates showed morphological differences regarding both curved size and growth. These differences may arise depending on environmental conditions at the sampling moment, laboratory procedures, and conservation techniques, among others [40, 41]. These strains presented positive activity for Voges–Proskeuer and nitrate reduction and negative activity against ONPG and H_2_S production.

The isolates BBCOL-026, BBCOL-027, and BBCOL-031 shared the main properties of the genus *Vibrio*. They were motile, curved, facultative, Gram-negative and oxidase-positive and were able to reduce nitrate to nitrite. These bacteria also formed yellow colonies, as reported by several authors [42, 43]. Optimal growth conditions for BBCOL-025 were at pH 6.0–6.5 and 37°C. The isolates showed high phenotypical homogeneity although variable reactions were observed for aesculin hydrolysis, *N*-acetyl-glucosaminidase activity, and fermentation of galactose and lactose.

The cells of strain BBCOL-032 *Staphylococcus* spp. were Gram-positive, motile, and nonsporulated, presenting an average bacterial size of 1.28 × 0.68 *µ*m. Cells were facultative and oxidase- and catalase-positive. Optimal growth conditions for BBCOL-025 were at pH 6.0–6.5 and 37°C. Isolated strains presented positive activity for the Voges–Proskauer test and nitrate reduction, and showed negative activity against ONPG and H_2_S production, as previously reported [44, 45].

### 3.3. Sodium Chloride and Perchlorate Susceptibility Assay

All isolates showed growth in the culture medium with high concentrations of NaCl, reaching tolerance up to 30%. Strains BBCOL-023 to BBCOL-033 presented characteristics of moderately halophilic bacteria, according to what was reported by Acevedo-Barrios [20].

The ability of isolates to grow and tolerate concentrations of KClO_4_ between 100 and 10000 mg/L is presented in Table 2. All isolates showed biofilm formation at the highest concentration. This barrier allows the bacteria to generate a concentration gradient as a means of protection against the toxicity of this chemical [2].

Other aspects evidenced in these strains are associations between NaCl and KClO_4_ tolerance. These isolates have become interesting targets for this research, given the need to identify native bacteria with potential biotechnological and biochemical versatility, capable of degrading environmental contaminants such as KClO_4_.

### 3.4. Evaluation of Perchlorate Reduction by Isolates

**Figure 7:**
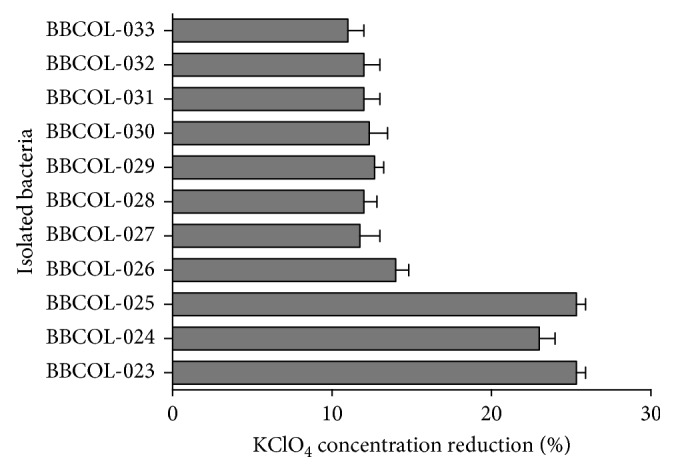
Percentage of KClO_4_ concentration reduction of bacteria BBCOL-023 to BBCOL-033 from saline environments in the Colombian Caribbean. Effect of the 48 h contact time, optical density at OD 600, and optimal pH (7.0 ± 0.5).

Perchlorate-reducing bacteria are phylogenetically diverse, and these include Alphaproteobacteria, Betaproteobacteria, Gammaproteobacteria, and Deltaproteobacteria classes, with Betaproteobacteria being the most commonly detected class [46, 47]. In this work, bacterial strains BBCOL-023 to BBCOL-033 (Figure 7) showed biological capacity to reduce concentrations of KClO_4_ on percentages between 10 and 25.

The genera *Nesiotobacter* and *Salinivibrio* showed the highest percentage (25%) of perchlorate reduction, while the genera *Vibrio*, *Bacillus*, and *Staphylococcus* presented a lowest proportion of KClO_4_ reduction, with 14, 12, and 10%, respectively. Recent studies have shown that the amount of perchlorate reduced may be inversely proportional to increased salinity [13, 17]. Future studies should be carried out to describe the role of salinity on perchlorate reduction by these strains.

The ability of bacteria to grow in perchlorate polluted areas is determined by their degrading enzymes [48, 49]. The general metabolic reduction pathway widely accepted by researchers [9, 10] involves the reductase enzyme, as this is responsible to reduce perchlorate to chlorate and chlorate to chlorite, while the superoxide chlorite enzyme changes chlorite to chloride and molecular oxygen [9, 46, 50]. The optimal temperature range for perchlorate reduction is 28–37°C [46, 51, 52].

Perchlorate-reducing bacteria are usually anaerobic and some facultative, despite being the molecular oxygen produced as an intermediate of the microbial perchlorate reduction [46, 53], in a process that exudes nitrate [46]. In our study, isolated perchlorate-reducing bacteria were also anaerobic but facultative. Therefore, although these may undergo degradation processes in a wide range of environmental conditions, it is also probable that some critical anaerobic strains were missed during the aerobic treatment. In consequence, additional experiments should be carried out under anaerobic conditions, just to enrich some active microorganisms that may improve perchlorate degradation.

Perchlorate is an ubiquitous and persistent pollutant in the environment, causing toxic effects in biota and humans. Therefore, different technologies have been developed to remove and eliminate this chemical. One of the most promising, effective, and economic ones is the use of bacteria in biotechnological systems that are capable of reducing and eliminating perchlorate. The rates of perchlorate reduction obtained in this study were comparable to those reported by Wang et al. [54], suggesting their potential application in bioremediation of perchlorate contaminated areas.

## 4. Conclusions

The strains isolated from Galerazamba-Bolivar, Manaure-Guajira, and Salamanca Island-Magdalena, Colombia, were halotolerant organisms belonging to the *Vibrio*, *Bacillus*, *Salinovibrio*, *Staphylococcus*, and *Nesiotobacter* genera. These strains could reduce KClO_4_ levels in aqueous solutions from 10 up to 25%. Bacteria-mediated remediation of perchlorate is a suitable process to control pollution by this toxic chemical.

## Figures and Tables

**Table 1:** Morphological and biochemical characteristics of isolated strains.

Characteristics	BBCOL-023	BBCOL-024	BBCOL-025	BBCOL-026	BBCOL-027	BBCOL-028	BBCOL-029	BBCOL-031	BBCOL-032	BBCOL-033
Molecular identification	*Nesiotobacter* sp.	*Bacillus vallismortis*	*Salinivibrio costicola*	*Vibrio alginolyticus*	*V*. *harveyi*	*B. cohnii*	*Bacillus* spp.	*Vibrio* sp.	*Staphylococcus* spp.	*B. flexus*
Color of colony	Beige	Yellow	Yellow	Yellow	Yellow	Yellow	Yellow	Yellow	Yellow	Yellow
Morphology	Rod shaped	Rod shaped	Vibrio shaped	Vibrio shaped	Vibrio shaped	Rod shaped	Rod shaped	Coco shaped	Rod shaped	Rod shaped
Length (*μ*m)	1.56	1.60	1.45	0.86	0.15	1.53	3.90	1.23	1.33	3.07
Thickness (*μ*m)	0.7	0.65	0.68	0.76	0.62	0.6	0.8	0.7	1.28	0.79
Motility	−	−	+	−	−	+	+	+	−	+
Gram straining	−	+	−	−	−	+	−	−	+	+
Endospore	−	+	−	−	−	+	−	−	−	+
Spore position	+	+	+	+	+	+	+	+	−	−
Oxidase	+	+	+	+	*W*	+	+	+	+	+
Catalase	−	+	−	−	−	+	+	−	+	+
Arabinose	−	+	+	+	+	+	+	+	+	+
Mannose	+	−	−	−	−	−	−	−	−	−
Sucrose	+	+	+	+	+	+	+	+	+	+
Melibiose	+	+	+	+	+	+	+	+	+	+
Rhamnose	+	+	+	+	+	+	+	+	+	+
Mannitol	−	−	+	+	+	+	+	+	+	+
Adonitol	−	−	−	−	−	−	−	−	−	−
Galactose	+	+	+	+	+	+	+	+	+	+
Inositol	−	−	−	−	−	−	−	−	−	−
p-n-p-Phosphate	−	−	+	−	+	−	−	−	+	−
p-n-p a-*ß*-Glucoside	*V*	−	−	−	+	−	*V*	−	−	−
p-n-p-*ß*-Galactoside	+	+	+	+	+	−	+	+	+	+
Prolinenitroanilide	−	−	−	−	+	−	−	−	−	−
p-n-p bis-Phosphate	−	−	−	−	−	−	−	−	−	−
p-n-p-Xyiloside	+	+	+	+	+	−	+	+	+	+
p-n-p-a-Arabinoside	+	+	+	+	+	−	+	+	+	+
p-n-p-Phosphorylcholine	−	−	−	−	−	−	−	−	−	−
p-n-p-*ß*-Glucuronide	−	−	−	−	−	−	−	−	−	−
p-n-p-*N*-Acetylglucosamide	−	−	−	+	+	−	−	+	−	−
*γ*-L-Glutamyl-p-nitroanilide	−	−	−	−	−	−	−	−	−	−
Aesculin	+	+	+	+	+	+	+	+	+	+
p-Nitro-DL-phenylalanine	−	−	−	−	−	−	−	−	−	−
Urea	−	−	−	−	−	−	−	−	−	−
Glycine	−	−	−	−	−	−	−	−	−	−
Citrate	*V*	−	*W*	+	+	−	*V*	−	*W*	+
Malonic acid	+	+	+	+	+	+	+	+	+	+
Triphenyltetrazoliumchloride	+	+	+	+	+	+	+	+	+	+
Lactose	+	+	+	+	+	+	+	+	+	+
Bacteriolytic capacity	+	+	+	+	+	+	+	+	+	+
Cellulolytic capacity	−	−	−	−	−	−	−	−	−	−
Nitrate reduction	+	+	+	+	+	+	+	+	+	+
Indole	−	−	−	−	−	−	−	−	−	−
ONPG	−	−	−	−	−	−	−	−	−	−
Ornithine utilization	−	−	−	−	−	−	−	−	−	−
H_2_S production	−	−	−	−	−	−	−	−	−	−
Voges–Proskauer's test	−	+	+	−	−	−	−	−	−	−
Methyl red	−	−	−	−	−	−	−	−	−	−
Sorbitol	+	−	−	−	−	−	−	−	−	−

+, positive reaction; −, negative reaction; *W*, weakly positive reaction; *V*, variable reaction.

**Table 2:** Bacterial strains BBCOL-023 to BBCOL-033 exposed to different NaCl and KClO_4_ concentrations and pH changes.

Characteristics	BBCOL-023	BBCOL-024	BBCOL-025	BBCOL-026	BBCOL-027	BBCOL-028	BBCOL-029	BBCOL-031	BBCOL-032	BBCOL-033
Maximum NaCl tolerance (%)	30	30	30	10	10	10	10	10	15	10
Maximum KClO_4_ tolerance (mg/L)	10,000	10,000	10,000	10,000	10,000	7,500	10,000	10,000	10,000	7,500
pH range tolerance	6.5–12	6.5–12	6.5–12	6.5–12	6.5–12	6.5–12	6.5–12	6.5–12	6.5–12	6.5–12

## Data Availability

The datasets generated and/or analyzed during the current study are available from the corresponding author on request.

## Abbreviations

LB NaCl: Luria-Bertani in seawater
ML: Maximum likelihood
MP: Maximum parsimony
ME: Minimum evolution
NJ: Neighbor joining
SEM: Scanning electron microscopy
OD: Optical density.
